# Assessment of CTX, PINP, and Vitamin D-Binding Protein in Gingival Crevicular Fluid and Saliva During Fixed Orthodontic Treatment

**DOI:** 10.3390/diagnostics16010030

**Published:** 2025-12-22

**Authors:** Ali Batuhan Bayırlı, Ebru Yurdakurban, Mehmetcan Uytun, Fulden Cantaş Türkiş, Ercan Saruhan

**Affiliations:** 1 Department of Periodontology, School of Dentistry, Mugla Sıtkı Kocman University, 48000 Mugla, Türkiye; mehmetcanuytun@mu.edu.tr; 2 Department of Orthodontics, School of Dentistry, Mugla Sıtkı Kocman University, 48000 Mugla, Türkiye; ebruyurdakurban@mu.edu.tr; 3 Department of Biostatistics, School of Medicine, Mugla Sıtkı Kocman University, 48000 Mugla, Türkiye; fuldencantas@mu.edu.tr; 4 Department of Biochemistry, School of Medicine, Mugla Sıtkı Kocman University, 48000 Mugla, Türkiye; ercansaruhan@mu.edu.tr

**Keywords:** fixed orthodontic treatment, gingival crevicular fluid, saliva, C-terminal telopeptide of type I collagen (CTX), procollagen type I N-terminal propeptide (PINP), vitamin D-binding protein, bone remodeling

## Abstract

**Background/Objectives**: Orthodontic tooth movement is a biological process involving coordinated bone resorption and formation in response to mechanical stimulation. The aim of this study was to evaluate the temporal changes in C-terminal telopeptide of type I collagen (CTX), procollagen type I N-terminal propeptide (PINP), and vitamin D-binding protein (VDBP) levels in gingival crevicular fluid (GCF) and saliva during fixed orthodontic treatment, as well as to assess the relationships among these biomarkers. **Methods**: The study included a total of 27 systemically and periodontally healthy individuals comprising 14 males and 13 females. Clinical periodontal parameters were assessed at three time points: before treatment (T0), at 24–48 h (T1), and on day 40 (T2). GCF and saliva samples were collected at the same time points. Levels of CTX, PINP and VDBP in GCF and saliva were quantified using enzyme-linked immunosorbent assay. The data were analyzed using both parametric and non-parametric statistical tests. Temporal changes across the three time points were evaluated using mixed-effects models, differences between GCF and saliva biomarker levels were assessed using paired tests, and correlations were examined using Spearman correlation analysis. **Results**: GCF and salivary CTX levels demonstrated a significant increase from T0 to T1, while PINP levels exhibited a substantial rise from T1 to T2 (*p* < 0.001). Levels of VDBP in both GCF and saliva did not demonstrate significant temporal changes (*p* > 0.05). Higher VDBP levels in both fluids were found to be negatively associated with increases in CTX and positively associated with increases in PINP (*p* < 0.05). Furthermore, salivary CTX and VDBP levels exhibited a consistent increase compared to those measured in GCF at all time points (*p* < 0.05). **Conclusions**: Fixed orthodontic forces elicit sequential resorptive and formative responses in both GCF and saliva. The potential of VDBP to function as a local modulator is indicated, with the capacity to influence the balance between osteoclastic and osteoblastic activity. The evaluation of these biomarkers in non-invasive biological samples may offer a valuable approach for monitoring bone metabolism throughout orthodontic treatment.

## 1. Introduction

The vitamin D-binding protein (VDBP) is a glycoprotein that is widely distributed in body fluids and organs. It plays a crucial role in vitamin D metabolism by binding to and transporting vitamin D and its metabolites. A positive correlation has been reported between serum VDBP concentrations and 1,25-dihydroxyvitamin D (1,25(OH)_2_D) levels [1]. The majority of circulating vitamin D is bound to VDBP, with a smaller portion bound to serum albumin, and only a minimal fraction existing in the free form. The VDBP demonstrates a high degree of affinity for 25(OH)D and a comparatively lower degree of affinity for 1,25(OH)_2_D [2]. The concentration of VDBP is significantly higher than the combined total of other forms of vitamin D [3]. With these characteristics, VDBP emerges as an ideal biomarker for assessing vitamin D levels in body fluids such as saliva and gingival crevicular fluid (GCF) [4,5]. Serum vitamin D levels have been reported to be associated with immune system regulation and bone remodelling metabolism, both of which play critical roles in the pathogenesis of periodontal diseases. Furthermore, individuals with vitamin D deficiency tend to exhibit increased severity of periodontal disease [6,7].

A healthy periodontium is clinically characterized by the absence of bleeding on probing, erythema, edema, clinical attachment loss, and alveolar bone resorption [8]. The radiographic evaluation process involves the assessment of the integrity of the lamina dura, as well as the distance from the alveolar bone crest to the cemento-enamel junction. In healthy individuals, this distance may vary depending on factors such as tooth morphology and root angulation [9]. The alveolar bone structure is influenced by various factors, including infections, bone metabolism disorders, medication use, hormonal changes, and mechanical stress. Among these, the force applied during orthodontic treatment represents one of the key forms of mechanical stress [10,11,12,13].

Orthodontic tooth movement (OTM) has been defined as a sterile inflammatory response that is triggered by mechanical force, leading to bone resorption and apposition in the alveolar bone [14,15,16]. The process involves the coordinated activity of osteoclasts, osteoblasts, immune cells, cytokines, and mechanosensory cells [17,18].

OTM consists of three distinct phases known as the initial, lag, and post-lag phases. Each of these phases is characterized by variable rates of bone resorption and formation. In the initial phase, the application of mechanical force is known to trigger the release of inflammatory mediators and stimulate osteoclastogenesis [19,20]. Neutrophils are the first cells to become activated during the initial phase of OTM, releasing chemotactic mediators that attract other immune cells. In the lag phase, necrosis occurs in the periodontal tissue on the compression side due to the applied orthodontic force, leading to a temporary halt in tooth movement. During this phase, macrophages clear the necrotic tissue, and both bone resorption and apposition are observed. In the post-lag phase, the inflammation persists as the activity of T and B cells increases, and proinflammatory cytokines continue to promote bone resorption [21,22].

During orthodontic treatment, cytokines and bone remodelling markers present in GCF and saliva have been shown to play a significant role in tooth movement [23,24,25,26]. One such marker is the C-terminal telopeptide of type I collagen (CTX), a biomarker released during the process of type I collagen degradation during bone resorption. The presence of this marker is indicative of the intensity of osteoclastic activity. Consequently, CTX functions as an indicator of bone resorption [26,27]. Procollagen type I N-terminal propeptide (PINP) has been demonstrated to play a role in osteoblastic activity. PINP is a biomarker that is released during the synthesis of type I collagen, specifically when procollagen is converted into fibrillar form. Consequently, it functions as an indicator of bone apposition [26,28].

Biochemical markers and underlying mechanisms are crucial in planning and regulating orthodontic treatment and understanding tooth movement. While the inflammatory mechanisms underlying OTM involve numerous cytokines, including interleukin-1β, interleukin-6, and tumour necrosis factor-α, these mediators were not included in the present investigation. Their exclusion reflects the methodological scope of this study, which was designed to examine bone remodelling responses rather than the broader immunoinflammatory network.

Throughout the course of orthodontic treatment, it is hypothesized that GCF and salivary VDBP, which show a correlation with serum vitamin D levels, may influence CTX and PINP levels. A review of the existing literature revealed no studies evaluating the levels of VDBP, PINP, and CTX in GCF and saliva during orthodontic treatment. Therefore, the present study aimed to investigate the changes in GCF and salivary CTX, PINP, and VDBP levels at different stages of conventional labial orthodontic treatment and to assess their interrelationships. In this context, the null hypothesis of the study is that VDBP, CTX, and PINP levels in GCF and saliva do not exhibit significant changes at different stages of orthodontic treatment, and that no significant relationships exist among these biomarkers.

## 2. Materials and Methods

### 2.1. Study Design and Participants

**Figure 1:**
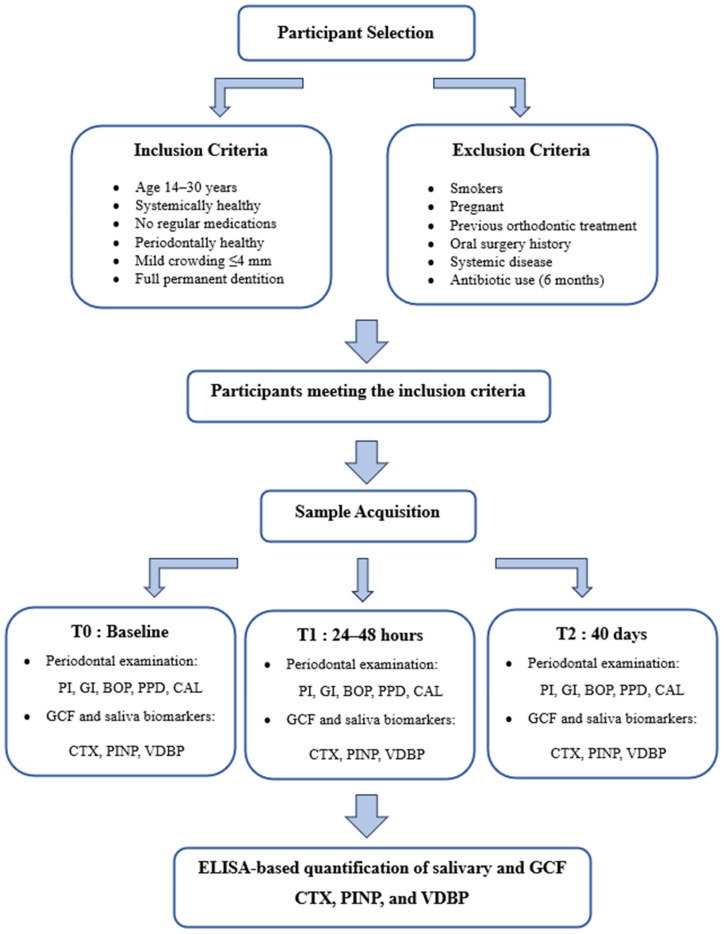
The flow chart of the study, including GCF and saliva sampling. Abbreviations: T0, baseline; T1, 24–48 h; T2, 40 days; GCF, gingival crevicular fluid; CTX, C-terminal telopeptide of type I collagen; PINP, procollagen type I N-terminal propeptide; VDBP, vitamin D-binding protein; PI, plaque index; GI, gingival index; BOP, bleeding on probing; PPD, probing pocket depth; CAL, clinical attachment loss.

This study was approved by the Non-Interventional Clinical Research Ethics Committee of İzmir Bakırçay University on 15 January 2025, under decision number 1972, and was conducted in accordance with the principles of the Declaration of Helsinki. All participants were thoroughly informed about the purpose and content of the study, and written informed consent was obtained from each individual. The study was conducted and reported in accordance with the Strengthening the Reporting of Observational Studies in Epidemiology (STROBE) guidelines. The clinical and radiographic examinations were performed at the Departments of Orthodontics and Periodontology, Faculty of Dentistry, Muğla Sıtkı Koçman University. Individuals meeting the following inclusion criteria were considered eligible for participation in the study: individuals aged between 14 and 30 years, those without any systemic diseases, individuals not using systemic or topical medications on a regular basis, periodontally healthy subjects, individuals presenting with mild crowding (≤4 mm) in both the maxillary and mandibular arches that did not require extraction, and individuals with complete permanent dentition. The following individuals were excluded from the study: smokers, pregnant individuals, those with a history of previous orthodontic treatment, individuals who had undergone surgical procedures involving soft or hard tissues in the maxillary or mandibular regions, those with any systemic diseases, and individuals who had used antibiotics within the past six months (Figure 1).

### 2.2. Study Groups

In order to ensure standardization, the study exclusively included individuals with moderate to mild crowding, ranging from 2 to 4 mm. For treatment standardization, 0.022-inch slot metal brackets (Dentsply GAC International, The Hauge, Netherlands) and 0.014-inch Sentalloy archwires (Dentsply GAC, New York, NY, USA) were used as the initial archwire for all patients. As part of the periodontal examination, the following indices were evaluated: plaque index (PI), gingival index (GI), bleeding on probing (BOP), probing pocket depth (PPD), and clinical attachment loss (CAL). The periodontal status of the participants in the study group was categorized according to the classification established jointly by the European Federation of Periodontology and the American Academy of Periodontology in 2017 [8]. The following criteria were utilized for the diagnosis of periodontal health: CAL ≤ 1 mm on all teeth, PPD ≤ 3 mm on all teeth, absence of radiographic bone loss, and BOP less than 10%.

Throughout the course of treatment, oral hygiene instructions were reinforced at each follow-up appointment, and professional plaque control was performed. Participants received detailed guidance on the proper use of manual toothbrushes, toothpaste, dental floss, and interdental brushes. All participants were advised to use orthodontic toothbrushes (STIM) twice daily. This standardized protocol aimed to minimize potential variations in clinical periodontal parameters among participants and thereby contributed to maintaining reliable and comparable conditions for salivary and GCF biomarker analyses.

### 2.3. Periodontal Examination

All participants underwent clinical and radiographic examinations to confirm periodontal health and to monitor any potential changes during the study period. Clinical measurements were recorded at six sites per tooth using a Williams periodontal probe (Hu-Friedy Manufacturing Co. Inc., Chicago, IL, USA). The examinations were carried out by two periodontologists under standardized conditions. Prior to the study, a calibration procedure was conducted on 10 subjects who were not included in the main study, with a view to ensuring measurement reliability. The clinical periodontal parameters were evaluated at three distinct time points: baseline (T0), 24–48 h (T1), and 40 days (T2).

### 2.4. Saliva Sample Acquisition

For the biochemical analysis of CTX, PINP, and VDBP levels, saliva samples were collected noninvasively using the passive drooling technique [29]. Samples were obtained from each participant at three different time points: T0, T1, and T2. To standardize the procedure and minimize biological variability, all samples were collected between 8:00 and 10:00 a.m. after an overnight fast into Eppendorf tubes. Female participants were scheduled outside the menstrual phase of their cycle to reduce potential hormonal effects on biomarker levels. Before providing 2–4 mL of saliva, participants were asked to rinse their mouths with water for two minutes to remove any food debris. The collected saliva samples were centrifuged at 1000× *g* for 10 min, and the supernatants were transferred into Eppendorf tubes and stored at a temperature of −80 °C until the biochemical analysis.

### 2.5. GCF Sample Acquisition

GCF samples were collected from the labial surfaces of the maxillary central incisors. Samples were obtained from each participant at three different time points: T0, T1, and T2. To ensure procedural consistency and minimize biological variability, all GCF samples were collected between 8:00 and 10:00 a.m. after an overnight fast. Female participants were scheduled outside the menstrual phase of their cycle to prevent hormonal influences on GCF biomarker levels.

For each participant, a single site from one tooth was used for sampling. Before GCF collection, supragingival plaque was carefully removed with a sterile curette without touching the gingival margin. The tooth was isolated using a cotton roll and a saliva ejector, then gently dried with a sterile 2 × 2 gauze pad. One paper strip was used per site. Standard-sized absorbent paper strips (2 mm × 14 mm, Periopaper; Proflow Inc., Amityville, New York, NY, USA) were inserted into the gingival sulcus and left in place for 30 s. Strips contaminated with saliva or blood were excluded from analysis. After collection, the paper strips were placed into Eppendorf tubes containing 300 μL of phosphate-buffered saline (PBS) and centrifuged at 1000× *g* for 10 min. The supernatants obtained from the GCF samples were separated for analysis, thoroughly mixed, and stored at −80 °C until the day of biochemical evaluation.

### 2.6. Biochemical Quantification of Salivary and GCF CTX Levels

The concentration of CTX in both saliva and GCF was measured using a human CTX enzyme-linked immunosorbent assay (ELISA) kit (Cat. No. E1349Hu, Bioassay Technology Laboratory, Shanghai, China) in accordance with the manufacturer’s instructions. The sensitivity of the CTX assay was 4.21 ng/mL. The intra- and inter-assay coefficients of variation of the assay were less than 10%. The measurement range for CTX was 7–1500 ng/mL.

### 2.7. Biochemical Quantification of Salivary and GCF PINP Levels

The concentration of PINP in both saliva and GCF was measured using a human PINP ELISA kit (Cat. No. E1350Hu, Bioassay Technology Laboratory, Shanghai, China) in accordance with the manufacturer’s instructions. The sensitivity of the assay was 2.51 ng/mL, and the intra- and inter-assay coefficients of variation were both below 10%. The measurement range for PINP was 5–2000 ng/mL.

### 2.8. Biochemical Quantification of Salivary and GCF VDBP Levels

The concentration of VDBP in both saliva and GCF was measured using a human VDBP ELISA kit (Cat. No. EA0045Hu, Bioassay Technology Laboratory, Shanghai, China) in accordance with the manufacturer’s instructions. The sensitivity of the assay was 0.021 µg/mL, and the intra- and inter-assay coefficients of variation were below 10%. The measurement range for VDBP was 0.05–20 µg/mL.

### 2.9. Statistical Analysis

The normality of data distribution was assessed using the Shapiro–Wilk test. Depending on the distributional characteristics and study design, appropriate parametric or non-parametric methods were applied. The analysis of differences across time points was conducted using the linear mixed-effects model, repeated-measures ANOVA, or Friedman test, as appropriate. Paired analyses were performed to compare dependent measurements between GCF and saliva using the paired *t*-test or Wilcoxon signed-rank test. Correlations between biochemical and clinical parameters were evaluated using Spearman’s rank correlation analysis, and the results were visualized through heatmaps. The data are presented as mean ± standard deviation (SD) or median (minimum–maximum), depending on the data distribution. All analyses were conducted using R software version 2025.09.0 (R Foundation for Statistical Computing, Vienna, Austria). A *p* value of <0.05 was considered statistically significant.

Power Analysis: The sample size was determined based on a power analysis performed with the G*Power 3.1.2 software (https://www.psychologie.hhu.de/arbeitsgruppen/allgemeine-psychologie-und-arbeitspsychologie/gpower, accessed on 26 October 2025), using the correlation coefficient reported in a comparable study in the literature [26]. An alpha level of 0.05 and a statistical power (1–β) of 0.80 were adopted in the analysis, which indicated that a minimum of 27 participants was required. Accordingly, the study was designed to include at least 27 individuals within a single cohort.

## 3. Results

A total of 27 periodontally healthy individuals were included in the study. The mean age of the participants was 17.6 ± 1.1 years, and the sample comprised 14 males and 13 females. The intra- and inter-examiner reproducibility of the periodontal measurements was assessed using the intraclass correlation coefficient (ICC). Intra-examiner ICC values were 0.94 (PPD) and 0.93 (CAL) for one examiner, and 0.92 (PPD) and 0.91 (CAL) for the other examiner, while inter-examiner ICC values were 0.91 (PPD) and 0.93 (CAL). Based on these results, the measurements were considered reliable. Evaluation of the clinical periodontal parameters measured at three different time points revealed no statistically significant changes in PI, GI, CAL, PD, or BOP scores (*p* > 0.05 for all comparisons). Similarly, analyses performed across sex and age groups showed no significant differences after adjustment for multiple comparisons (*p* > 0.05) (Table 1).

Significant temporal changes were observed in specific biochemical parameters in both GCF and saliva. PINP levels showed statistically significant differences across the three time points in both GCF and saliva (*p* < 0.001). Pairwise comparisons revealed no significant difference between T0 and T1 (*p* > 0.05), whereas a statistically significant increase was observed from T1 to T2 in both sample types (*p* < 0.001 for both). Similarly, CTX levels measured in both GCF and saliva demonstrated statistically significant differences across time (*p* < 0.001). Pairwise comparisons showed a statistically significant increase in CTX levels from T0 to T1 in both GCF and saliva (*p* < 0.001 for both), whereas the changes observed between T1 and T2 were not statistically significant (*p* > 0.05). For VDBP, no statistically significant differences were detected across the three time points in either GCF or saliva (*p* > 0.05). Consistently, pairwise comparisons also revealed no significant changes in VDBP levels (*p* > 0.05) (Table 2).

Significant differences were observed between GCF and saliva for several biochemical parameters at all time points. At T0, salivary CTX levels were significantly higher than those measured in GCF (557.47 ± 259.43 pg/mL and 454.52 ± 81.43 pg/mL, respectively; *p* = 0.033). Salivary VDBP concentrations were also markedly elevated compared with GCF values (5.66 [0.91–8.62] and 2.04 [0.94–3.97], respectively; *p* < 0.001). In contrast, GCF PINP levels were significantly greater than those detected in saliva (820.46 ± 245.73 ng/mL and 643.70 ± 338.05 ng/mL, respectively; *p* = 0.014). At T1, the similar pattern observed at baseline persisted, with salivary CTX levels remaining significantly higher than those in GCF (916.33 ± 395.46 pg/mL and 641.48 ± 116.75 pg/mL, respectively; *p* < 0.001). Salivary VDBP concentrations were also markedly elevated compared with GCF values (5.15 ± 2.39 and 2.23 ± 1.03, respectively; *p* < 0.001). In contrast, PINP levels continued to be significantly higher in GCF than in saliva (827.52 ± 249.05 ng/mL and 667.17 ± 300.65 ng/mL, respectively; *p* = 0.018). At T2, both CTX and VDBP concentrations were again significantly greater in saliva than in GCF (*p* < 0.001). However, no significant difference in PINP levels was observed between the two sample types at this time point (*p* = 0.269) (Table 3).

**Figure 2:**
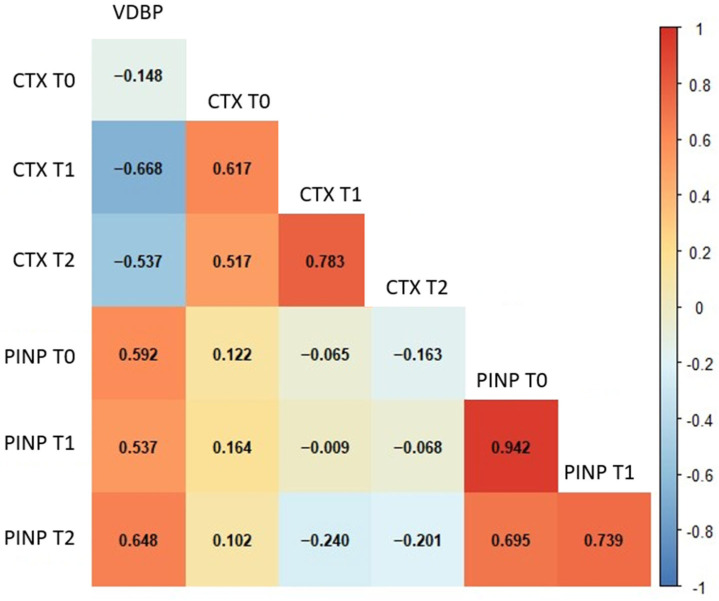
Spearman’s correlation heatmap of GCF biochemical parameters across different time points. The heatmap illustrates the strength and direction of correlations among GCF parameters. Red and blue colours represent positive and negative correlations, respectively. Statistically significant correlations (*p* < 0.01) are indicated by stronger colour intensity. Abbreviations: GCF, gingival crevicular fluid; VDBP, vitamin D-binding protein; CTX, C-terminal telopeptide of type I collagen; PINP, procollagen type I N-terminal propeptide; T0, baseline; T1, 24–48 h; T2, 40 days.

**Figure 3:**
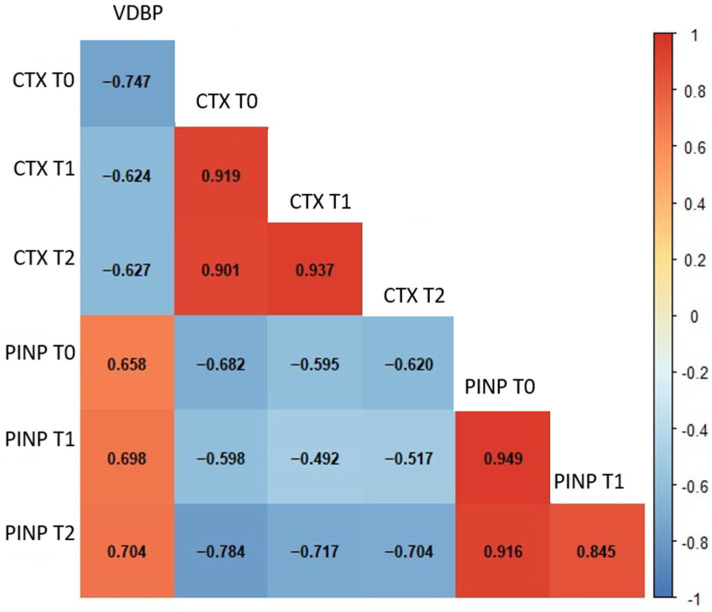
Spearman’s correlation heatmap of salivary biochemical parameters across different time points. The heatmap illustrates the strength and direction of correlations among salivary parameters. Red and blue colours represent positive and negative correlations, respectively. Statistically significant correlations (*p* < 0.01) are indicated by stronger colour intensity. Abbreviations: VDBP, vitamin D-binding protein; CTX, C-terminal telopeptide of type I collagen; PINP, procollagen type I N-terminal propeptide; T0, baseline; T1, 24–48 h; T2, 40 days.

Considering the results of the correlation analysis, several meaningful associations were identified among the biochemical parameters across different time points in GCF. VDBP T0 levels showed a moderate negative correlation with CTX at T1 (*p* < 0.001) and CTX at T2 (*p* = 0.004). In contrast, VDBP T0 was positively correlated with PINP levels at all time points, including PINP T0 (*p* = 0.001), PINP T1 (*p* = 0.004), and PINP T2 (*p* < 0.001) (Figure 2). Strong and coherent correlation patterns were observed among the salivary biochemical parameters (Figure 3). Baseline VDBP (T0) levels demonstrated strong negative correlations with CTX across all time points (all *p* < 0.001). In contrast, VDBP T0 was positively correlated with PINP values at all measurement times (all *p* < 0.001).

In GCF, CTX levels were significantly influenced by time (*p* < 0.001), VDBP levels (*p* = 0.005), and their interaction (*p* < 0.001). CTX concentrations increased markedly at both T1 and T2 relative to baseline, while higher VDBP levels were associated with a smaller increase over time. For GCF PINP, both time and VDBP were significant predictors (*p* < 0.05). PINP levels increased at T2, and higher VDBP concentrations were associated with greater PINP levels. In saliva, CTX was significantly affected by time and VDBP (both *p* < 0.01), whereas the interaction term was not significant. CTX increased at both follow-up points, consistent with a negative association with VDBP. For salivary PINP, VDBP and its interaction with time were significant determinants (both *p* < 0.01), indicating that higher VDBP levels corresponded to increased PINP concentrations, particularly at T2 (Table 4).

## 4. Discussion

The present study evaluated changes in the bone metabolism–related biomarkers CTX, PINP, and VDBP in both GCF and saliva among periodontally healthy individuals undergoing orthodontic treatment. The absence of significant alterations in clinical periodontal parameters across the three time points indicates that the applied orthodontic forces did not compromise periodontal tissues. Previous clinical research assessing periodontal responses over the early stages of orthodontic treatment, typically at baseline, one month and three months, has shown that clear aligner therapy maintains periodontal stability, whereas fixed appliances lead to greater plaque accumulation and early gingival inflammation [30]. In a longitudinal study, periodontal status was assessed at baseline and at the one-month follow-up using PI and GI, and the findings indicated that fixed orthodontic appliances do not induce pronounced periodontal alterations within the first month of treatment [31]. This limited early change aligns with the stability of periodontal parameters observed during the 40-day follow-up period in our study. In a clinical study, fixed orthodontic treatment was shown to produce a statistically significant increase in PPD within the first month, while CAL remained largely unchanged [32]. However, in our study, the absence of early periodontal changes did not preclude the detection of notable biochemical responses. The significant variations observed in CTX and PINP levels demonstrate that orthodontic forces directly influence bone metabolism and that these biochemical responses occur independently of inflammation, driven primarily by mechanical stimulation.

The findings of this study demonstrate that orthodontic force application influences bone resorption and formation in a sequential manner. The significant increase in CTX levels in both GCF and saliva from T0 to T1 suggests early osteoclastic activation and the onset of collagen degradation in the alveolar bone. The absence of further changes in CTX between T1 and T2 indicates that resorptive activity may reach a plateau phase. These results align with the role of CTX as a specific biochemical marker of bone resorption and are consistent with previous reports describing the early phase of orthodontic tooth movement as a period characterized by osteoclast differentiation and the initiation of bone resorption on the compression side [19,20,33]. Other research has demonstrated that peri–miniscrew crevicular fluid shows no significant change in CTX levels at 24 h following orthodontic force application, but exhibits a marked increase by day seven [34]. Similarly, other research has shown a significant rise in GCF CTX-I levels during the fourth week of continuous orthodontic loading [35]. These temporal differences may reflect variations in experimental conditions, duration of force application, or the biological dynamics of tissue response, suggesting that CTX elevation may occur at different phases depending on the magnitude and continuity of the applied orthodontic force.

Another key finding of our study involves the changes in PINP levels, a marker of bone formation that follows the resorptive phase. The absence of significant differences in GCF and salivary PINP levels between T0 and T1 indicates that bone formation does not begin immediately after orthodontic force application and that the early response progresses primarily through the resorptive phase. The marked increase observed from T1 to T2 reflects enhanced osteoblastic activity and increased collagen synthesis. These results are consistent with previous reports demonstrating that osteoblastic bone formation follows osteoclastic resorption during the second phase of bone remodelling induced by orthodontic force [19,20,36].

During this period, the ability of mechanical stimulation to regulate osteogenic signaling pathways in bone cells and to promote osteoblast differentiation and new bone matrix synthesis may explain the observed increase in PINP levels [37,38]. The sequential alterations in CTX and PINP levels reflect the physiological mechanism of orthodontic tooth movement at the biochemical level and demonstrate that the alveolar bone adapts to the applied mechanical forces. These findings contrast with other research that demonstrated no significant temporal changes in serum and GCF CTX and PINP levels in patients undergoing fixed orthodontic treatment [26]. This discrepancy may be attributed to differences in sampling time points and the type of biological fluid analyzed. Serum, as a systemic biological medium, may be insufficient to capture localized bone remodeling responses. Conversely, the GCF and saliva samples utilized in this study may offer a more direct and sensitive reflection of the biochemical changes occurring within the periodontal microenvironment.

Following the sequential biochemical responses associated with bone resorption and formation, the absence of significant differences in VDBP levels across the three time points suggests that short-term orthodontic loading does not substantially influence vitamin D metabolism or the dynamics of its carrier proteins. VDBP, which serves as the primary transport protein in vitamin D metabolism, binds approximately 85% of circulating 25(OH)D and 1,25(OH)_2_D, thereby maintaining their serum concentrations within physiological limits [39]. This high binding capacity may explain why no significant changes in serum VDBP levels were observed during short-term orthodontic force application. Other research has also found an association between VDBP levels in saliva and orthodontic tooth movement, with levels remaining stable within an optimal range [4]. Excessively low or high levels, however, reduce the amount of tooth movement. When considered alongside the results of the present study, this finding suggests that VDBP may be a stable biomarker for short-term orthodontic forces. However, the existing literature offers inconsistent findings regarding the effect of vitamin D on bone metabolism during orthodontic treatment. While some studies suggest that vitamin D3 may promote bone formation and accelerate tooth movement by enhancing osteoblast activity via the RANKL/OPG axis, others indicate that the correlation between serum or saliva vitamin D levels and bone biomarkers is minimal [17,40].

To the best of our knowledge, no previous study has simultaneously evaluated the relationship between VDBP levels and CTX and PINP in both GCF and saliva. The findings obtained in the correlation analysis revealed that baseline VDBP levels were negatively correlated with CTX and positively correlated with PINP in both GCF and saliva samples, suggesting that this protein may play different roles in bone resorption and formation processes. These results suggest that VDBP may be associated with the dynamic phases of bone metabolism in the local bone microenvironment, rather than merely acting as a carrier protein. Mixed-effects model analyses also supported these relationships; when the time factor was considered, high VDBP levels were found to be inversely related to CTX increases and directly related to PINP increases.

These parallel findings suggest that VDBP levels in both gingival crevicular fluid and saliva may indirectly contribute to the balance of osteoclastic and osteoblastic activities during the orthodontic loading process. Therefore, this relationship between VDBP and changes in the local bone microenvironment can be considered a complementary marker in understanding the biochemical responses observed during orthodontic tooth movement. Similarly, other research demonstrated that salivary VDBP levels remained within an optimal range during orthodontic tooth movement, where both low and high concentrations were associated with reduced tooth displacement [41]. These findings are consistent with our observations, suggesting that the bidirectional associations of VDBP with resorptive (CTX) and formative (PINP) markers may reflect its modulatory role in maintaining the local balance between osteoclastic and osteoblastic activity during orthodontic loading.

Significant biochemical differences were observed when comparing sample types between GCF and saliva. At all time points, CTX and VDBP levels in saliva were consistently higher than in GCF. This finding indicates that these parameters reflect the systemic bone cycle rather than local periodontal remodelling. However, higher PINP concentrations were detected in GCF during the initial phase (T0–T1), indicating a more pronounced local osteoblastic response within the periodontal microenvironment. The disappearance of the difference in PINP levels between the two biological fluids at T2 may reflect a transition in bone formation dynamics from a local to a systemic process as the orthodontic adaptation process progresses. The literature also reports that GCF primarily reflects local tissue remodelling processes, whereas saliva reflects both local and systemic biochemical responses [4,24,25,26].

These divergent patterns may also reflect the broader cytokine and mediator networks operating differentially within GCF and saliva, involving regulatory pathways that extend beyond the specific biomarkers assessed in the present study. Saliva and GCF are known to contain locally concentrated inflammatory and osteogenic mediators that respond rapidly to mechanical loading and closely mirror periodontal ligament remodelling [42,43]. In contrast, saliva represents a more integrated biological fluid influenced by systemic circulation, salivary gland secretion, and mucosal transudate, which can dilute or obscure localized cytokine fluctuations. Previous studies have shown that cytokine interactions within these two fluids do not always follow parallel trajectories, owing to differences in molecular origin, transport dynamics, and enzymatic degradation patterns, supporting the notion that they reflect biologically distinct compartments rather than methodological variability [44,45]. Therefore, the discrepancies observed between GCF and saliva in our study are most plausibly attributable to intrinsic physiological differences between these biofluids.

Taken together, these biological distinctions provide a basis for interpreting the differential biomarker patterns observed in our study. These differences reveal that GCF and saliva reflect distinct yet complementary aspects of bone metabolism. Therefore, the findings suggest that different biological samples may provide complementary information regarding bone metabolism markers and that VDBP in particular may play a bridging role in this interaction. Furthermore, measuring VDBP in GCF and saliva, compared to assessing serum vitamin D levels, offers a more non-invasive approach that prioritizes patient comfort in clinical practice. It may also be considered a promising biomarker candidate for future studies monitoring bone metabolism during orthodontic treatment.

The primary strength of this study is that the evaluation of these three biomarkers, which reflect bone remodelling during fixed orthodontic treatment, was conducted simultaneously and at multiple time points in both GCF and saliva samples. This prospective, repeated-measurement design enabled the monitoring of dynamic changes in bone resorption and formation following orthodontic loading over time. Selecting participants who were periodontally healthy and free of systemic disease, with mild to moderate crowding, reduced potential confounding factors and ensured that the biochemical responses obtained could be attributed more specifically to orthodontic forces. Furthermore, using two different biological sample types (GCF and saliva) simultaneously allows for complementary interpretation of the local periodontal microenvironment and broader oral/systemic biochemical responses. Furthermore, this study is one of the first to suggest that monitoring VDBP levels locally during fixed orthodontic treatment may be clinically significant in maintaining healthy periodontal tissue. From a clinical perspective, the observed temporal patterns and the inverse correlations between VDBP and CTX suggest that combined assessment of these biomarkers in GCF and saliva may provide a non-invasive approach for monitoring bone resorption dynamics during orthodontic treatment. Such biomarker-based monitoring could help clinicians better understand individual biological responses to orthodontic forces and potentially support more personalized treatment strategies.

Additionally, smokers and individuals who had recently undergone periodontal or oral surgical procedures were excluded because these conditions are known to alter local inflammatory and microbiological profiles, potentially affecting the baseline or temporal expression of bone turnover markers in GCF and saliva. Smoking influences vascularity and immune responses, while recent soft- or hard-tissue surgical interventions may induce transient microbiological shifts even when adjunctive laser-assisted decontamination is performed [46,47]. These changes could confound the biomarker kinetics evaluated in the present study.

However, certain limitations should also be considered. Firstly, the study was single-centre, with short-term follow-up and a relatively small sample size. Therefore, caution should be exercised when generalizing the results to different age groups, more severe types of malocclusion or different orthodontic treatment protocols. The study did not evaluate serum 1,25(OH)_2_D or VDBP levels. Another limitation is that local findings could not be directly correlated with systemic vitamin D status. Furthermore, potential confounding variables affecting bone metabolism, such as body mass index, physical activity level, sun exposure and craniofacial morphology, were not controlled for. Outcome variables such as the amount of orthodontic tooth movement and clinical tooth movement rate were not evaluated alongside biomarker profiles, which also limits the direct correlation of the findings with clinical outcomes. Previous research indicates that insufficient sun exposure and low serum vitamin D levels may alter bone turnover dynamics, potentially influencing orthodontic tooth movement patterns [40,48]. Likewise, variations in the amount or rate of tooth movement have been associated with differences in local bone remodelling responses [49]. These observations suggest that such parameters could further refine the interpretation of biochemical markers in future studies. Future studies may also investigate potential strategies to reduce microbiological and biological variability in GCF and saliva during OTM. Adjunctive approaches such as laser photobiomodulation could theoretically stabilize local tissue responses, whereas systemic agents including vitamin D supplementation, anti-inflammatory medications or host-modulation therapies may influence bone turnover dynamics or modify the oral microbiome. However, these interactions have not yet been elucidated, representing an important area for future research. Despite these limitations, the present study may offer an early indication of a potential bidirectional relationship between VDBP and the bone turnover markers CTX and PINP in both GCF and saliva. These findings may provide a preliminary foundation for future research involving larger samples, extended observation periods, and the assessment of systemic levels.

## 5. Conclusions

The findings of this study reveal that the fixed orthodontic treatment process leads to sequential and dynamic changes in biochemical markers reflecting bone remodelling. The increase observed in CTX levels in the early period indicates the onset of the resorptive phase, while the subsequent increase in PINP indicates the activation of the formation phase. Although short-term orthodontic force did not cause a significant change in VDBP concentrations, the negative correlation of VDBP with CTX and positive correlation with PINP suggests that this protein may be a local modulator regulating the balance between osteoclastic and osteoblastic activity. The simultaneous evaluation of GCF and saliva reflected different but complementary aspects of bone metabolism. Consequently, the assessment of CTX, PINP, and VDBP in non-invasive biological samples such as GCF and saliva may offer a clinically valuable approach for monitoring bone metabolic responses during orthodontic tooth movement.

## Figures and Tables

**Table 1:** Changes in clinical periodontal parameters over time.

	T0	T1	T2	$\chi^{2}$/F	*p*
PI	0.72 (0.55–0.98)	0.72 (0.55–0.98)	0.72 (0.55–0.98)	0.252	0.881
GI	0.58 (0.27–0.86)	0.58 (0.27–0.86)	0.58 (0.27–0.80)	2.787	0.248
CAL (mm)	0.60 (0.38–0.86)	0.53 (0.38–0.94)	0.51 (0.38–0.91)	1.021	0.600
BOP (%)	7.67 (5.83–9.94)	7.75 (4.72–9.69)	7.94 (4.72–9.97)	0.804	0.669
PPD (mm)	1.63 ± 0.26	1.62 ± 0.27	1.64 ± 0.25	0.026	0.974

Values are presented as median (min–max) or mean ± standard deviation, as appropriate. Friedman test and repeated-measures ANOVA were used to assess within-group differences, with χ^2^ and F statistics reported, respectively. Abbreviations: PI, plaque index; GI, gingival index; PPD, probing pocket depth; CAL, clinical attachment loss; BOP, bleeding on probing; T0, baseline; T1, 24–48 h; T2, 40 days.

**Table 2:** Changes in GCF and salivary biochemical parameters over time.

	T0	T1	T2	$\chi^{2}$	*p*
**GCF**					
CTX (ng/mL)	454.52 ± 81.43 ^a^	641.48 ± 116.75 ^b^	621.30 ± 102.91 ^b^	90.621	**<0.001**
VDBP (ng/mL)	2.04 (0.94–3.97)	2.05 (0.81–3.96)	2.09 (0.87–4.06)	3.185	0.203
PINP (ng/mL)	820.46 ± 245.73 ^a^	827.52 ± 249.05 ^a^	1138.03 ± 200.93 ^b^	59.112	**<0.001**
**Saliva**					
CTX (ng/mL)	557.47 ± 259.43 ^a^	916.33 ± 395.46 ^b^	884.84 ± 325.56 ^b^	76.076	**<0.001**
VDBP (ng/mL)	5.27 ± 2.47	5.15 ± 2.39	5.19 ± 2.36	1.027	0.365
PINP (ng/mL)	699.80 (142.70–241.20) ^a^	712.90 (172.60–1220) ^a^	1240.70 (255.80–1564) ^b^	37.630	**<0.001**

Values are presented as median (min–max) or mean ± standard deviation, as appropriate. Friedman test and repeated-measures ANOVA were used to assess within-subject differences, with χ^2^ and F statistics reported, respectively. Identical superscript letters within the same row indicate no significant difference between time points, whereas different letters denote statistically significant differences (*p* < 0.05). Abbreviations: GCF, gingival crevicular fluid; CTX, C-terminal telopeptide of type I collagen; PINP, procollagen type I N-terminal propeptide; VDBP, vitamin D-binding protein; T0, baseline; T1, 24–48 h; T2, 40 days.

**Table 3:** Comparison of biochemical parameters between GCF and saliva at each time point.

		CTX	VDBP	PINP
T0	GCF	454.52 ± 81.43	2.04 (0.94–3.97)	820.46 ± 245.73
	Saliva	557.47 ± 259.43	5.66 (0.91–8.62)	643.70 ± 338.05
	t/Z	−2.246	−4.493	2.638
	*p*	**0.033**	**<0.001**	**0.014**
T1	GCF	641.48 ± 116.75	2.23 ± 1.03	827.52 ± 249.05
	Saliva	916.33 ± 395.46	5.15 ± 2.39	667.17 ± 300.65
	t/Z	−4.110	−9.016	2.516
	*p*	**<0.001**	**<0.001**	**0.018**
T2	GCF	621.30 ± 102.91	2.09 (0.87–4.06)	1200 (795.20–1446)
	Saliva	884.84 ± 325.56	5.53 (0.77–9.41)	1240.70 (255.80–1564)
	t/Z	−4.792	−4.469	−1.105
	*p*	**<0.001**	**<0.001**	0.269

Values are presented as median (min–max) or mean ± standard deviation, as appropriate. Paired *t*-test or Wilcoxon signed-rank test was applied to compare GCF and salivary concentrations for each parameter at corresponding time points. Statistically significant differences are shown in bold (*p* < 0.05). Abbreviations: GCF, gingival crevicular fluid; CTX, C-terminal telopeptide of type I collagen; PINP, procollagen type I N-terminal propeptide; VDBP, vitamin D-binding protein; T0, baseline; T1, 24–48 h; T2, 40 days.

**Table 4:** Fixed-effects estimates from linear mixed-effects models for CTX and PINP levels in GCF and saliva.

	Fixed Effects	β	SE_β_	t	*p*	Model Fit (R^2^m/R^2^c)	Significant ANOVA Terms
**CTX GCF**	(Intercept)	473.56	37.65	12.58	**<0.001**	0.562/0.860	Time (*p* < 0.001), VDBP (*p* = 0.005), Time × VDBP (*p* < 0.001)
	Time (T1)	309.82	31.61	9.80	**<0.001**		
	Time (T2)	268.77	31.13	8.64	**<0.001**		
	VDBP	–8.52	15.10	–0.56	0.576		
	Time × VDBP T1	–55.15	12.84	–4.29	**<0.001**		
	Time × VDBP T2	–45.11	12.52	–3.60	**<0.001**		
**PINP GCF**	(Intercept)	629.02	92.63	6.79	**<0.001**	0.407/0.793	Time (*p* = 0.002), VDBP (*p* = 0.013)
	Time T1	29.44	80.97	0.36	0.718		
	Time T2	278.59	79.75	3.49	**0.001**		
	VDBP	85.63	37.18	2.30	**0.026**		
	Time × VDBP T1	–9.79	32.90	–0.30	0.767		
	Time × VDBP T2	16.45	32.07	0.51	0.610		
**CTX saliva**	(Intercept)	916.51	121.40	7.55	**<0.001**	0.422/0.888	Time (*p* < 0.001), VDBP (*p* = 0.001)
	Time T1	410.05	79.48	5.16	**<0.001**		
	Time T2	306.73	80.26	3.82	**<0.001**		
	VDBP	–68.14	20.73	–3.29	**0.002**		
	Time × VDBP T1	–11.52	13.90	–0.83	0.411		
	Time × VDBP T2	2.96	14.01	0.21	0.834		
**PINP saliva**	(Intercept)	217.51	116.23	1.87	0.068	0.552/0.894	VDBP (*p* < 0.001), Time × VDBP (*p* = 0.004)
	Time T1	51.48	83.16	0.62	0.539		
	Time T2	165.97	83.97	1.98	0.054		
	VDBP	80.88	19.90	4.07	**<0.001**		
	Time × VDBP T1	–3.56	14.54	–0.25	0.807		
	Time × VDBP T2	43.78	14.65	2.99	**0.004**		

Values represent estimated regression coefficients (β) with corresponding standard errors (SE_β_), t statistics, and *p* values derived from linear mixed-effects models. Random intercepts were included to account for within-subject variability. R^2^m indicates the marginal R^2^ (variance explained by fixed effects), and R^2^c denotes the conditional R^2^ (variance explained by both fixed and random effects). Significant ANOVA terms summarize the overall effects of time, vitamin D, and their interaction on each biochemical parameter. Statistically significant predictors are shown in bold (*p* < 0.05). Abbreviations: GCF, gingival crevicular fluid; CTX, C-terminal telopeptide of type I collagen; PINP, procollagen type I N-terminal propeptide; VDBP, vitamin D-binding protein; T1, 24–48 h; T2, 40 days.

## Data Availability

The data presented in this study are available on request from the corresponding author due to ethical and privacy.
